# Young children’s perceptions of health warning labels on cigarette packages: a study in six countries

**DOI:** 10.1007/s10389-014-0612-0

**Published:** 2014-02-26

**Authors:** Dina L. G. Borzekowski, Joanna E. Cohen

**Affiliations:** 1 Department Behavioral and Community Health, School of Public Health, University of Maryland, College Park, MD 20742 USA; 2 Johns Hopkins Bloomberg School of Public Health, Baltimore, MD USA

**Keywords:** Warning labels, Cigarettes, Tobacco, Global, Awareness

## Abstract

**Aim:**

Health warning labels on cigarette packages are one way to reach youth thinking about initiating tobacco use. The purpose of this study was to examine awareness and understanding of current health warning labels among 5 and 6 year old children.

**Subjects and methods:**

Researchers conducted one-on-one interviews with urban and rural 5 and 6 year olds from Brazil, China, India, Nigeria, Pakistan, and Russia.

**Results:**

Among the 2,423 participating children, 62 % were unaware of the health warnings currently featured on cigarette packages, with the lowest levels of awareness in India and the highest levels in Brazil. When shown the messages, the same percentage of participating children (62 %) showed no level of message understanding.

**Conclusion:**

While youth are receiving social and informational messages promoting tobacco use, health warning labels featured on cigarette packages are not effectively reaching young children with anti-smoking messages.

## Introduction

Globally, over 1 billion people smoke with the majority (>80 %) living in low- and middle-income countries (Jha et al. 2002). Every year, tobacco kills nearly 6 million people and it accounts for 10 % of all adult deaths (Shafey et al. 2009). Interventions that decrease tobacco uptake and use by youth can greatly reduce tobacco-related disease and disabilities (Koh et al. 2011).

Typically, smoking behaviors are established during adolescence (Global Youth Tobacco Survey Collaborative Group 2002; Gilpin and Pierce 1997) and those who begin smoking before age 13 are twice as likely to become regular adult smokers, compared to those who begin smoking at age 17 or later (Breslau and Pereson 1996). Experimentation and initiation with one’s first cigarette may occur during early primary grades, with greater risks for those who have family members who smoke and for those who have household access to cigarettes (Leonardi-Bee et al. 2011; Gilpin et al. 2004). Additionally, young children are familiar with tobacco brands, advertising and promotions (Borzekowski and Cohen 2013); these marketing efforts are associated with early experimentation and smoking in adulthood (Gilpin et al. 2007; DiFranza et al. 2006; Emria et al. 1998).

Health warnings featured on cigarette packaging may be one effective way to reach children with anti-smoking messages, especially when family and friends smoke cigarettes and cigarette packages are commonplace items in the household. Under the WHO’s Framework Convention on Tobacco Control (FCTC), parties are required to adopt and implement measures to create packaging and labeling that effectively communicate the health risks associated with tobacco use (WHO 2008). While they may not be the primary target audience of warning labels, elementary school-aged children are certainly one of the secondary audiences of these labels. According to the Guidelines for Article 11 of the FCTC, youth are identified as a population subgroup to be reached with such health warnings (WHO 2008).

While health warnings currently vary in terms of positioning, coverage, and strength, there are studies that show among adolescent and adult samples that labels can communicate health information, alter risk perceptions, and prevent smoking initiation (Hammond 2011). Besides being able to recall these messages, smokers and non-smokers indicate that these warnings are important information sources (Shanahan and Elliott 2009). Recent findings from ITC-4 data (a four-country policy evaluation study) showed that the warning messages had a protective effect against relapse in ex-smokers 1 year after quitting (Partos et al. 2012). The same data also added to evidence that warning labels increase quitting attempts (Borland et al. 2009). Among adult non-smokers, graphic messages conveying high risk were salient, and discouraged smoking initiation (Kessels 2012; Shanahan and Elliott 2009).

Warning labels, especially those with large graphic pictures, are effective in reaching ‘low-literate populations, children and young people’; well-designed health warnings are more likely to be noticed and can better communicate health risks (WHO 2008; Fong 2007). The literature suggests that messages appealing to negative emotions or fear are more effective (Hammond 2011). Despite arguments suggesting that gruesome imagery may have the unintended effect of viewers suppressing the conveyed message, the association with displeasure is said to influence attitudes about cigarette use (Selin 2009). Furthermore, a study of message framing in warning labels in Canada found that both smoking and non-smoking adolescents were more likely to avoid smoking from the negatively framed messages, and also perceived them as more effective (Goodall 2008). The same study found that labels depicting an older person were less effective than those that featured a close-up of decaying teeth. Another study showed that graphic images of either short-term cosmetic or long-term health effects were effective message themes for adolescents (Smith 2003).

Children as young as 5 and 6 years old, frequently encounter and are aware of social and environmental cues encouraging the use of tobacco products (Borzekowski and Cohen 2013; Freeman et al. 2005), but little evidence exists on whether very young children are exposed to and understand messages about smoking’s harmful effects. One multi-country study recently described that the majority (87.6 %) of adolescent youth (ages 13–15 years) were exposed to counter-marketing and supported smoke-free policies (Koh et al. 2011); however, literature on younger children and their perceptions does not appear to exist. Ideally, it would be best for young children to avoid encountering cigarette packages, but, given that these are familiar objects in children’s informational environments, such packages should effectively communicate health warnings and messages.

Critical to the success of health communication initiatives are basic awareness and understanding. A range of factors related to both the audience and the messaging do have an impact, influencing whether one sees and comprehends messaging (Wilson 2007). Petty and Cacioppo’s (1986) theory of persuasion, known as the Elaboration Likelihood Model (ELM), can be used to explain how different people process and engage in the presented material. ELM considers whether message receivers not only attend to different messages but also if they are capable of understanding them. Source and message factors come into play such as where and how messages are delivered and if the messages use a fear appeal (Wilson 2007). Audience and information processing factors underlie effective messaging that may discourage smoking initiation among children (Peracchio and Luna 1998). Working with 7 and 8-year-old American youth, researchers have found that only the most straightforward messages are understood (Peracchio and Luna 1998). While counter-marketing and health warnings about smoking are being communicated to varying degrees in different countries (Shafey et al. 2009), we are unaware of any other international research examining very young children’s awareness and understanding of current messages discouraging the use of tobacco. It is critical to know about youth exposure to and comprehension of such messages, especially in the years prior to when they are tempted to initiate smoking.

Conducted in 2012 in six countries—Brazil, China, India, Nigeria, Pakistan, and the Russian Federation (hereto forth referred to as Russia)—this study’s purpose was to examine young children’s awareness and understanding of health warnings on cigarette packaging. This analysis explores whether demographic variables, social exposure to smoking, awareness of tobacco brands, and intentions to smoke were associated with awareness and understanding of health warnings. Information from this type of work can inform tobacco control interventions including message development for counter-marketing campaigns as well as future health warnings.

This study had two main outcomes, awareness and understanding of health warnings. The research questions explored by this study included: (1) Are young children aware of the warning labels on cigarette packages?; (2) Do young children understand the warning label messages on cigarette packages?; and, (3) What variables, including demographics, household smoking status, familiarity with tobacco brands, and intentions to smoke are significantly associated with awareness of and understanding of warning labels on cigarette packages?

## Methods

The World Health Organization (WHO) divides the world’s countries into six groups: the Region of the Americas, the South-East Asia Region, the Western Pacific Region, the Eastern Mediterranean Region, the African Region, and the European Region. This study was done in the low or middle-income country with the highest number of smokers in each region: Brazil, China, India, Pakistan, Nigeria, and Russia. As of November 2010, the current adult tobacco smoking rates for men and women, respectively, were 22 and 13 % in Brazil, 53 and 2 % in China, 24 and 3 % in India, 9 and 0.2 % in Nigeria, 32 and 6 % in Pakistan, and 60 and 22 % in Russia (WHO 2012). Also, it should be noted that 33 and 18 % of Indian men and women and 34 and 6 % of Pakistani men and women are current users of smokeless tobacco (WHO 2012).

Regarding warning labels, the countries varied in their policies and implementation (Hammond 2013). In Brazil, policies on health warning labels on cigarette packages were implemented in 2002. There, warnings must cover 100 % of either the front or back of the package. Every few years, a set of ten new warnings are developed and introduced. The Brazilian policy also bans the use of misleading terms such as “light” and “mild” on cigarette packages. In China, pictorial warnings are used on promotional material but only in Hong Kong and Macau are there requirements for picture warnings on cigarette packages. Small text warnings have appeared on Chinese cigarette packages until October 2008, when they were increased to cover 30 % of the front and back surface (Fong et al. 2010). In India, a policy for health warnings was drafted in 2006; two warnings were released in 2008 and started appearing on packages in 2009. In India, warnings are required to cover 40 % of the front of cigarette packages. Nigeria uses only one image on cigarette packages, and the coverage requirement is 43 % on both the front and back of the package. In 2010, Pakistan passed legislation to have cigarette packages display picture warnings that cover 40 % of both the front and back. Russia passed regulations on pictorial health warnings in 2012, and these were to be implemented in 2013. Text messages in Russian of “smoking kills” are required to cover 30 % of the front of cigarette packages. A rotation of 12 text messages covering 50 % of the back of packages was the existing policy as of 2012, and new requirements will incorporate a series of 12 picture-based warnings covering 50 % of the back of the package with a text message remaining on 30 % of the front of the packages.

In each of the six countries, the research team worked with in-country public health professionals to select locations, focusing on residential areas of low- and middle-income households, that would clearly represent an urban and a rural population. Table 1 provides information on the geographic areas from which each sample was drawn. A cluster sample strategy was performed where the populations of low- and middle-income regions were first identified and then neighborhoods for recruitment were randomly selected. In India, Nigeria, Pakistan and Russia, researchers went on a specified path through a neighborhood and found households where either a 5- or 6-year-old lived and where there was a parent or guardian available to give consent. In Brazil and China, the population of schools where 5- and 6-year olds were in attendance was identified. Letters were sent home to all eligible students, asking parents if they and their children would be willing to participate in a health survey. From those willing to participate, researchers randomly selected subjects and came to the schools on consecutive mornings and afternoons to interview children and their parents/guardians. Data on eligible participants and refusals by country are available upon request. In each country, official in-country review boards approved the study design and protocols. Additionally, overall review and approval was obtained through the Johns Hopkins Institutional Review Board.

**Table 1:** Information about the sample (*N* = 2423)

Data collection locations	Overall		Brazil: around and near Rio de Janeiro		China: around and near towns of the Shanxi Province		India: around and near New Delhi		Nigeria: around and near Ile-Ife, in the Osun State		Pakistan: around and near Islamabad and Rawapindi		Russia: around and near Moscow and Nizhniy Novgorod	
	*N*	%	*N*	%	*N*	%	*N*	%	*N*	%	*N*	%	*N*	%
Gender														
Male	1,260	52.0	183	46.0	204	51.5	260	58.6	193	50.1	219	54.9	201	50.1
Female	1,163	48.0	215	54.0	192	48.5	184	41.4	192	49.9	180	45.1	200	49.9
Age														
5 years	1,119	46.2	169	42.5	152	38.4	224	50.5	195	50.7	179	44.9	200	49.9
6 years	1,304	53.8	229	57.5	244	61.6	220	49.5	190	49.3	220	55.1	201	50.1
Location														
Rural	1,228	50.7	198	49.7	198	50.0	222	50.0	189	49.1	186	46.6	200	49.9
Urban	1,195	49.3	200	50.3	198	50.0	222	50.0	196	50.9	213	53.4	201	50.1
Household tobacco users														
None	1,582	65.5	320	81.4	115	29.1	355	80.3	373	97.6	199	49.9	218	54.4
One or More	832	34.5	73	18.6	280	70.9	87	19.7	9	2.3	200	50.1	183	45.6
Familiarity with tobacco brands														
None	777	32.1	162	40.7	56	14.1	107	24.1	188	48.8	64	16.0	200	49.9
One or More	1,646	67.9	236	59.3	340	85.9	337	75.9	197	51.2	335	84.0	201	50.1
Intentions to smoke														
No	2,096	86.5	366	92.0	310	78.3	310	69.8	343	89.6	378	94.7	387	96.5
Yes	327	13.5	32	8.0	86	21.7	134	30.2	40	10.4	21	5.3	14	3.5

One of the authors personally trained local researchers to use the instruments, ensuring standardization but allowing for cultural variations across countries. As an example of cultural variation, we were advised to remove, and did remove, any questions about alcohol brands and use in Pakistan. Active oral consent was used and one-on-one interviews with the parent and child lasted around 8 and 30 min, respectively. Additionally, pilot testing was done in each country to test the feasibility of the instruments in terms of whether they could be understood and/or easily manipulated by the child. The parent and child interviews usually occurred simultaneously and children were always able to see their parent or guardian, but researchers tried to position the child so that the child’s responses could not be heard or observed by the parent. The child instrument started with demographics followed by questions asking about media use, intentions, attributes of a smoker, logo picture identification, food preferences, and lastly, warning labels. Data collection was conducted in the spring, summer and fall of 2012.

### Measures

#### Warning labels

**Fig. 1:**
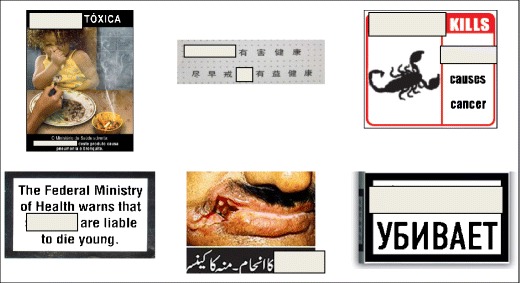
Examples of current health warning labels featured on cigarette packages, as examined in this study (*Top row*: Brazil, China, India;* Bottom row*: Nigeria, Pakistan, and Russia). As shown, we blanked out any words related to smoking or tobacco when we showed these warnings to the participating children

In five of the countries, preschool children were presented with two separate images of current health warning labels (from their own countries), with the words “smoking” or “tobacco” blanked out. Selection of warning labels was done by in-country teams and reflected current and popular labels. When there were options, teams picked warning labels that were not overly inappropriate (i.e., warnings discussing impotency) or gruesome (i.e., warnings showing a gangrenous foot) since we were working with young children. In China and Nigeria, just one image was used and each featured just text (see Fig. 1 for examples of health warnings shown to participating children). After being asked “have you ever seen this before,” children were encouraged to tell the researcher what the image was about. Responses were coded 0: no understanding; 1: weak understanding; and 2: solid understanding. To get a score of 1, the child had to mention something related to tobacco use or something related to harm or illness. A score of 2 required a response mentioning something related to *both tobacco use and harm*.

#### Demographics

This study collected information on demographics and household environment from the participating parents/guardians. In addition to asking questions about the gender, age, education, household resources, and smoking behaviors of all the people living in the household, researchers asked the parent/guardian about the child’s gender, and age and they asked the participating children if and how frequently they attended school.

#### Familiarity with tobacco brands

Children played a matching game to show familiarity with brands and their respective objects. In this game, there were 24 brand logos of which eight were tobacco brands (four domestic and four international brands). Researchers ensured that children knew what the objects were and then laid out the object cards so the children could physically handle them. Among the object cards, there were foils not represented by any brand logos (i.e., sneakers, tea, automobiles) and pictures of question marks, so children could indicate when they did not know a brand logo. The researchers then presented eight pages of logos (one page at a time, each with three logos per page). Children were instructed to place an object card in the box next to the logo that it represented. Children received scores for their familiarity with tobacco brands; for this analysis, we use the dichotomous variable of “none” or “one or more.”

#### Intentions to smoke cigarettes

Researchers handed the children a Yes/No card and presented a series of nine questions about who they might be and what they might do when they grew up and were “big people.” Children could point to either the “yes” or the “no.” Among the statements such as “Do you think you will drive a car?” and “Do you think you will be in trouble with the police?”, was a question that asked “do you think you will smoke cigarettes? Children’s responses were coded and each child had either a “no” or “yes” regarding having an intention to smoke cigarettes.

### Statistical analyses

After preliminary exploration of the data, we mainly used bivariate analyses (chi-square tests) to examine factors associated with awareness of and understanding of the health warnings. Among the independent variables we tested were child’s gender, age, school attendance, home location, household tobacco users, familiarity with tobacco brands, and intentions to smoke. We also created more complex multivariate models predicting awareness and understanding (both as dichotomous outcomes). The aforementioned variables were included in the models, excluding school attendance because this one variable was greatly skewed (85.4 % said they attended school) and not statistically significant in the one sample with less skew (Russia). For all analyses, Stata 11 software was used (StataCorp 2009).

## Results

**Fig. 2:**
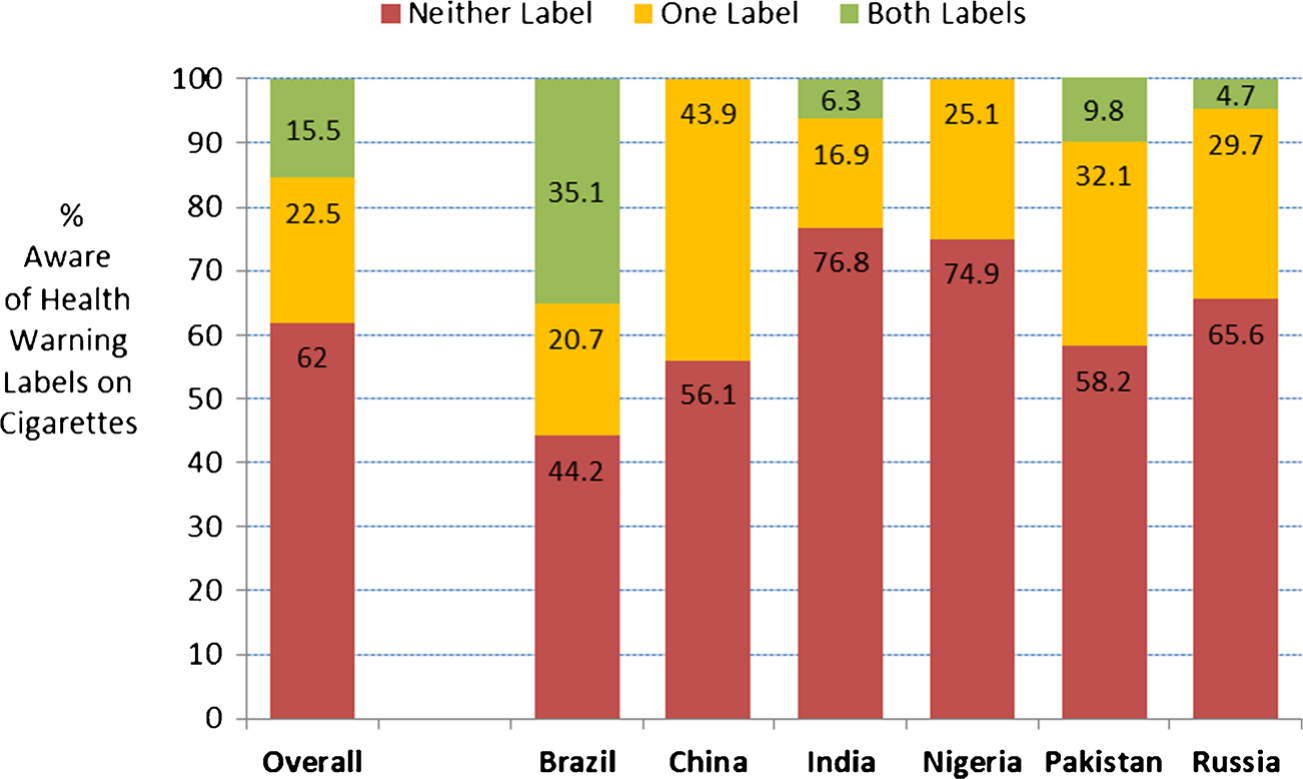
Percentage of children who were aware of the health warning labels on cigarette packages, by country. Note: Only one label was shown to the children in China and Nigeria

Over 2,400 5 and 6 year olds, and one parent or guardian for each, participated in this study. Information about the sample is presented in Table 1. While close to a third (34.5 %) of the children had one or more tobacco users in their households and two-thirds (67.9 %) were familiar with one or more of the tobacco brand logos, most children interviewed in this study were unaware of health warnings on cigarette packages. As shown in Fig. 2, 62.4 % were unaware of the labels that were currently being used in their countries. The most aware children were in Brazil, where 35.1 % indicated they had seen both labels. The least aware were in India and Nigeria, where 76.8 and 74.9 %, respectively, said they had not seen either label.

Table 2 offers information on variables that were (and were not) significantly associated with awareness of health warning labels. Other than in Pakistan, gender was not significantly associated with awareness. Being slightly older was related to awareness, overall (*Χ* ^2^ = 33.7, *p* < 0.001) and in China, India, and Nigeria (see Table 2 for country statistics). A child’s home location was associated with awareness—overall, urban children were more familiar with health warning labels (*Χ* ^2^ = 9.4, *p* < 0.01), but the relationship varied by country. In China and Nigeria, rural children were more familiar with the labels, while in Russia, urban children were more likely to know one or more of the labels in comparison to rural children. Living in a household where there was one or more smokers was associated with awareness (overall,* Χ* ^2^ = 38.5, *p* < 0.01). Almost twice as many children living with a tobacco user knew warning labels (21.5 %) compared to those living in a household with no tobacco users (12.3 %). Overall, 6 % of the children who did not know any tobacco brands were aware of both health warning labels, compared to 20 % who knew one or more brands (*Χ* ^2^ = 90.6, *p* < 0.001). This association was significant in all countries except India. A child’s intention to smoke as an adult was associated with awareness (*Χ* ^2^ = 7.6, *p* < 0.05). In China, slightly more than 60 % of those with intentions to smoke knew the warning label in contrast to 39.4 % of those without intentions to smoke.

**Table 2:** Children’s awareness of health warning labels

	Brazil			China		India			Nigeria		Pakistan			Russia		
	*N* = 398			*N* = 396		*N* = 444			*N* = 329		*N* = 399			*N* = 401		
	None	One	Both	No	Yes	None	One	Both	No	Yes	None	One	Both	None	One	Both
Gender																
Male	45.1	23.6	31.3	55.4	44.6	73.1	19.2	7.7	76.0	24.0	54.3	37.4	8.2	68.7	26.4	5.0
Female	43.5	18.2	38.3	56.8	43.2	82.1	13.6	4.4	73.8	26.2	62.8	25.6	11.6	62.5	33.0	4.5
	NS			*NS*		NS			NS		*Χ* ^2^ = 6.8, *p* < 0.05			NS		
Age																
5 Years	50.0	20.2	29.8	68.4	31.6	83.9	11.6	4.5	70.1	29.9	62.0	29.1	8.9	51.0	33.0	16.0
6 Years	39.9	21.1	39.0	48.4	51.6	69.6	22.3	8.2	79.9	20.1	55.0	34.6	10.5	39.3	36.3	24.2
	NS			*Χ* ^2^ = 15.3, *p* < 0.001		*Χ* ^2^ = 12.9, *p* < 0.01			*Χ* ^2^ = 4.9, *p* < 0.05		NS			NS		
Location																
Rural	44.9	21.9	33.2	39.9	60.1	77.9	15.4	6.8	67.5	32.5	61.5	31.5	7.0	72.6	21.9	5.5
Urban	43.5	19.5	37.0	72.2	27.8	75.7	18.5	5.9	82.4	17.5	54.3	32.8	12.9	58.5	37.5	4.0
	NS			*Χ* ^2^ = 57.4, *p* < 0.001		NS			*Χ* ^2^ = 11.5, *p* < 0.001		NS			*Χ* ^2^ = 11.7, *p* < 0.01		
Household tobacco users																
No	48.4	21.7	29.9	51.3	48.7	76.1	18.9	5.1	75.1	24.9	69.4	16.1	14.6	67.4	25.7	6.9
Yes	26.0	16.4	57.5	57.9	42.1	79.3	9.2	11.5	66.7	33.3	65.0	18.0	17.0	63.4	34.4	2.2
	*Χ* ^2^ = 20.5, *p* < 0.001			NS		*Χ* ^2^ = 8.5, *p* < 0.05			NS		NS			*Χ* ^2^ = 7.4, *p* < 0.05		
Familiarity with tobacco brands																
None	62.1	18.6	19.3	76.8	23.2	80.4	17.8	1.9	79.8	20.2	75.0	23.4	1.6	73.0	25.0	2.0
One or More	31.9	22.1	46.0	52.7	47.3	75.7	16.6	7.7	70.3	29.7	54.9	33.7	11.3	58.2	34.3	7.5
	*Χ* ^2^ = 39.7, *p* < 0.001			*Χ* ^2^ = 11.4, *p* = 0.001		NS			*Χ* ^2^ = 4.6, *p* = 0.031		*Χ* ^2^ = 10.8, *p* = 0.003			*Χ* ^2^ = 12.6, *p* = 0.002		
Intentions to smoke																
No	43.8	21.4	34.8	60.6	39.4	74.8	17.1	8.1	77.3	22.7	66.9	17.2	15.9	65.4	29.7	4.9
Yes	48.4	12.9	38.7	39.5	60.5	81.3	16.4	2.2	55.0	45.0	71.4	14.3	14.3	71.4	28.6	0.0
	NS		*Χ* ^2^ = 12.2, *p* < 0.001			NS			*Χ* ^2^ = 9.5, *p* < 0.01		NS			NS		
Total	44.2	20.7	35.1	56.1	43.9	76.8	16.9	6.3	74.9	25.1	58.2	32.1	9.8	65.6	29.7	4.7

**Fig. 3:**
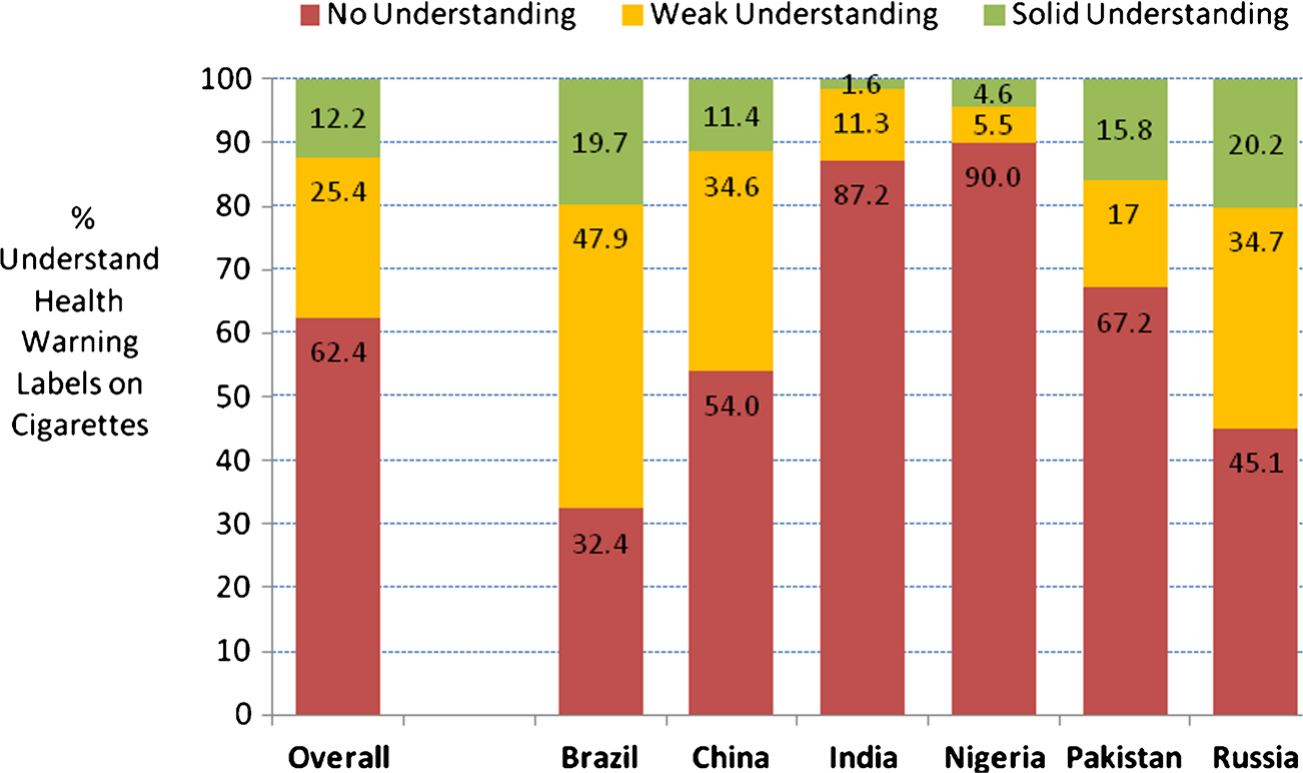
Percentage of children who understood the health warning labels on cigarette packages by country

Very few (12.2 %) of the children across the six country samples had solid understanding of existing health warning labels on cigarette packages. The majority, 62.4 %, had no understanding (see Fig. 3). The greatest percentage of no understanding was in Nigeria, closely followed by India. The most solid understanding was in Russia and Brazil.

Overall, across the six countries, all the considered variables were significantly associated with better understanding—gender (boys more than girls), *Χ* ^2^ = 6.6, *p* < 0.05; older age, *Χ* ^2^ = 53.5, *p* < 0.001; home location (rural more than urban), *Χ* ^2^ = 7.0, *p* < 0.05; living with a tobacco user, *Χ* ^2^ = 31.9, *p* < 0.001; familiarity with cigarette brands, *Χ* ^2^ = 37.3, *p* < 0.001; and smoking intentions *Χ* ^2^ = 10.8, *p* < 0.01 (Table 3). In Pakistan, girls were less likely than boys to understand the warning labels. Being 6 compared to being 5 years old was associated with greater understanding in all countries except Nigeria. In both Brazil and China, higher percentages of rural children compared to urban children gave a solid description of the warning labels. Overall, and in every country except for India, there was a significant relationship between familiarity with cigarette brands and understanding of warning labels. A child’s intentions to smoke as an adult were associated with understanding, but varied in direction. In China, higher percentages of those with intentions to smoke, compared to those without intentions, had solid understanding. In contrast, lower percentages of Indian children with smoking intentions had solid understanding compared to those without smoking intentions.

**Table 3:** Children’s understanding of cigarette warning labels

	Brazil			China			India			Nigeria			Pakistan			Russia		
	*N* = 398			*N* = 396			*N* = 444			*N* = 329			*N* = 399			*N* = 401		
	None	Weak	Solid	None	Weak	Solid	None	Weak	Solid	None	Weak	Solid	None	Weak	Solid	None	Weak	Solid
Gender																		
Male	27.1	53.6	19.3	52.0	36.8	11.3	85.0	13.1	1.9	90.7	4.3	4.9	62.6	21.5	16.0	41.8	39.3	18.9
Female	36.9	43.0	20.1	56.3	32.3	11.5	90.2	8.7	1.1	89.2	6.6	4.2	72.8	11.7	15.6	48.5	30.0	21.5
	NS			NS			NS			NS			*Χ* ^2^ = 7.1, *p* < 0.05			NS		
Age																		
5 Years	36.5	51.5	12.0	73.7	16.5	9.9	91.1	6.7	2.2	92.6	4.3	3.1	73.7	13.4	12.9	51.0	33.0	16.0
6 Years	29.4	45.2	25.4	41.8	45.9	12.3	83.2	15.9	1.0	87.4	6.6	6.0	61.8	20.0	18.2	39.3	36.3	24.2
	*Χ* ^2^ = 11.2, *p* < 0.01			*Χ* ^2^ = 41.6, *p* < 0.001			*Χ* ^2^ = 10.4, *p* < 0.01			NS			*Χ* ^2^ = 6.4, *p* < 0.05			*Χ* ^2^ = 6.8, *p* < 0.05		
Location																		
Rural	20.5	54.9	24.6	37.9	48.5	13.6	90.5	8.6	1.0	91.7	3.6	4.8	70.4	14.1	15.5	47.8	35.3	16.9
Urban	44.0	41.0	15.0	70.2	20.7	9.1	84.8	14.0	2.3	88.2	7.5	4.5	63.4	20.4	16.1	42.5	34.0	23.5
	*Χ* ^2^ = 25.4, *p* < 0.001			*Χ* ^2^ = 43.0, *p* < 0.001			NS			NS			NS			NS		
Household tobacco users																		
No	34.7	50.8	14.5	52.2	37.4	10.4	87.9	10.1	2.0	90.0	5.3	4.7	69.4	16.1	14.6	47.3	30.7	22.0
Yes	20.6	35.6	43.8	54.6	33.6	11.8	83.1	16.1	0.0	88.9	11.1	0.0	65.0	18.0	17.0	42.6	39.3	18.0
	*Χ* ^2^ = 32.1, *p* < 0.001			NS			NS			NS			NS			NS		
Familiarity with tobacco brands																		
None	41.0	49.7	9.3	83.9	16.1	0.0	91.6	5.6	2.8	95.3	4.1	0.6	81.3	9.4	9.4	55.5	27.5	17.0
One or More	26.5	46.6	26.9	49.1	37.7	13.2	85.8	13.1	1.2	84.3	6.9	8.8	64.5	18.5	17.0	34.8	41.8	23.4
	*Χ* ^2^ = 21.4, *p* < 0.001			*Χ* ^2^ = 24.7, *p* < 0.001			NS			*Χ* ^2^ = 14.5, *p* < 0.001			*Χ* ^2^ = 6.9, *p* = 0.035			*Χ* ^2^ = 17.4, *p* < 0.001		
Intentions to smoke																		
No	32.4	47.5	20.1	57.1	34.5	8.4	84.2	13.9	1.9	89.5	5.8	4.8	66.9	17.2	15.9	45.2	34.4	20.4
Yes	32.3	51.6	16.1	43.0	34.9	22.1	94.0	5.2	0.8	94.1	2.9	2.9	71.4	14.3	14.3	42.9	42.9	14.3
	NS			*Χ* ^2^ = 13.6, *p* < 0.001			*Χ* ^2^ = 8.1, *p* < 0.05			NS			NS			NS		
Total	32.4	47.9	19.7	54.0	34.6	11.4	87.2	11.3	1.6	90.0	5.5	4.6	67.2	17.0	15.8	45.1	34.7	20.2

A child’s awareness of the labels was significantly associated with their understanding of the health warning labels across all the countries (*Χ* ^2^ = 773.7, *p* < 0.001), and in each country sample (Brazil *Χ* ^2^ = 126.8, *p* < 0.001; China *Χ* ^2^ = 236.6, *p* < 0.001; India *Χ* ^2^ = 130.4, *p* < 0.001, Nigeria *Χ* ^2^ = 53.6, *p* < 0.001, Pakistan *Χ* ^2^ = 164.4, *p* < 0.001, and Russia *Χ* ^2^ = 153.5, *p* < 0.001). Among those with no awareness (had seen neither warning label), only 35 children (1.5 % of the entire sample) could provide a solid explanation of the label. Of those who were aware of both labels, only a third (33.9 %) had solid understanding of what these warnings were about.

Tables 4 and 5 offer multivariate models and indicate, if considered together, this set of variables predict both awareness and understanding respectively. For the overall dataset, having any awareness was more likely for older participants who lived in a household where someone used tobacco, and had familiarity with tobacco brands and logos. In China and Nigeria, having an intention to smoke in the future was predictive of having a higher likelihood of awareness of warning labels. Predicting understanding in the pooled data set, girls were less likely than boys to exhibit understanding; however, the Brazilian sample may be influencing this finding. Older children and those who lived in households with someone who used tobacco were more likely to convey some knowledge about the warning labels. Awareness of the labels, but not of cigarette brands, was predictive of greater understanding. Finally, in the overall data set, those with intentions to smoke as adults were less likely to exhibit understanding of the labels. Considering country data sets, we only observed intentions to smoke to be significant in India.

**Table 4:** Logistic regression examining factors associated with any awareness of warning labels (*N* = 2,423)

	Overall Adj. odds ratio (95 % CI)	Brazil Adj. odds ratio (95 % CI)	China Adj. odds ratio (95 % CI)	India Adj. odds ratio (95 % CI)	Nigeria Adj. odds ratio (95 % CI)	Pakistan Adj. odds ratio (95 % CI)	Russia Adj. Odds Ratio (95 % CI)
Sex							
Male	1.00	1.00	1.00	1.00	1.00	1.00	1.00
Female	0.97 (0.82, 1.15)	0.85 (0.55, 1.31)	0.85 (0.55, 1.33)	0.58 (0.36, 0.93)	1.18 (0.72, 1.92)	0.74 (0.49, 1.13)	1.39 (0.91, 2.14)
Age							
5 years	1.00	1.00	1.00	1.00	1.00	1.00	1.00
6 years	1.37 (1.15, 1.62)	1.33 (0.86, 2.05)	2.33 (1.44, 3.76)	2.32 (1.46, 3.70)	0.58 (0.35, 0.96)	1.28 (0.84, 1.94)	0.96 (0.63, 1.46)
Location							
Urban	1.00	1.00	1.00	1.00	1.00	1.00	1.00
Rural	1.14 (0.96, 1.35)	1.19 (0.77, 1.84)	3.50 (2.25, 5.44)	0.84 (0.53, 1.33)	1.96 (1.18, 3.23)	0.67 (0.44, 1.01)	0.54 (0.35, 0.83)
Someone in the child’s household uses tobacco	1.25 (1.05, 1.49)	2.00 (1.10, 3.63)	0.75 (0.46, 1.22)	0.80 (0.44, 1.46)	0.84 (0.19, 3.79)	1.52 (1.00, 2.30)	1.05 (0.68, 1.61)
Familiarly with cigarette brands	1.93 (1.59, 2.33)	3.29 (2.10, 5.16)	2.62 (1.27, 5.37)	1.49 (0.85, 2.62)	1.61 (0.98, 2.66)	2.12 (1.14, 3.94)	1.94 (1.26, 3.00)
Intention to smoke	1.02 (0.80, 1.31)	0.88 (0.40, 1.93)	2.42 (1.41, 4.16)	0.73 (0.43, 1.22)	2.30 (1.12, 4.69)	0.74 (0.28, 1.93)	0.72 (0.21, 2.46)

**Table 5:** Factors associated with the understanding of warning labels (*N* = 2,423)

	Overall Adj. odds ratio (95 % CI)	Brazil Adj. odds ratio (95 % CI)	China Adj. odds ratio (95 % CI)	India Adj. odds ratio (95 % CI)	Nigeria Adj. odds ratio (95 % CI)	Pakistan Adj. odds ratio (95 % CI)	Russia Adj. odds ratio (95 % CI)
Sex							
Male	1.00	1.00	1.00	1.00	1.00	1.00	1.00
Female	0.80 (0.65, 0.97)	0.59 (0.36, 0.97)	0.51 (0.27, 1.00)	0.78 (0.38, 1.62)	0.85 (0.36, 2.03)	0.70 (0.40, 1.22)	0.66 (0.42, 1.05)
Age							
5 years	1.00	1.00	1.00	1.00	1.00	1.00	1.00
6 years	1.72 (1.40, 2.11)	1.20 (0.74, 1.95)	4.85 (2.34, 10.08)	1.26 (0.62, 2.55)	2.62 (1.06, 6.45)	1.85 (1.05, 2.25)	1.77 (1.13, 2.79)
Location							
Urban	1.00	1.00	1.00	1.00	1.00	1.00	1.00
Rural	1.20 (0.98, 1.47)	4.20 (2.47, 7.15)	2.40 (1.25, 4.62)	0.39 (0.19, 0.79)	0.27 (0.11, 0.69)	0.76 (0.44, 1.33)	1.12 (0.71, 1.78)
Someone in the children’s household uses tobacco	1.52 (1.23, 1.88)	1.37 (0.69, 2.73)	1.18 (0.58, 2.39)	2.37 (1.02, 5.49)	2.19 (0.21, 22.91)	0.85 (0.48, 1.49)	1.00 (0.63, 1.59)
Awareness of warning labels							
None	1.00	1.00	1.00	1.00	1.00	1.00	1.00
One or both	11.48 (9.35, 14.08)	4.37 (2.64, 7.23)	50.46 (24.70, 103.08)	22.27 (10.50, 47.24)	24.34 (8.66, 68.41)	24.58 (13.53, 44.64)	9.10 (5.24, 15.80)
Familiarly with cigarette brands	1.10 (0.88, 1.38)	2.10 (1.23, 3.61)	4.25 (1.49, 12.09)	1.94 (0.80, 4.71)	3.42 (1.29, 9.09)	1.43 (0.60, 3.37)	1.92 (1.22, 3.04)
Intention to smoke	0.56 (0.41, 0.76)	1.14 (0.48,2.75)	1.00 (0.45, 2.21)	0.32 (0.13, 0.77)	0.34 (0.07, 1.81)	1.11 (0.30, 4.07)	1.26 (0.37, 4.36)

## Discussion

Among the 5 and 6 year olds in this international sample, there were low levels of awareness and understanding of the health warnings featured on cigarette packages. Half to three-quarters of the within country samples were not at all familiar with labels currently featured on tobacco products. In general, those who were slightly older, residing in rural areas, living with tobacco users, and familiar with tobacco brands and logos were more aware of the labels. Girls, 6 year olds, those living with tobacco users, and those more familiar with tobacco brands also had greater understanding of the health warning labels (although, for understanding the gender effect, this was observed only in Pakistan). Interestingly, the highest awareness and understanding was in Brazil where graphic health warning labels have been featured since 2002 and cover 100 % of the back of each cigarette package (Thrasher et al. 2010).

This is an important preliminary study in considering the reach of health warning labels to young children. While great effort was made to use developmentally, culturally appropriate, and current measures, drawing from health communication work with children (Borzekowski et al. 2013; Borzekowski and Macha 2010), the sample was very young and naïve to the research process. It was unlikely that any participants had ever been interviewed or asked their perceptions about environmental messages. Some children may have been trying to please the researchers, others may have been intimidated. Consequently, the presented awareness rates might be inflated as the questions only required the child to respond “no” or “yes” if she had seen the warning label. In contrast, the understanding rates may be underestimates, as this measure asked children to verbalize their responses (mentioning two aspects about the label to get a solid understanding score). Possibly, children may have known more about warning labels but were not able to articulate their knowledge. To further examine the internal and external validity of awareness and understanding, future studies would need more involved approaches, perhaps over time. One idea might be to explore natural environments and observe what messages are truly in the children’s homes and communities. Another idea is to have a child explain to a peer the pro- and anti-messages she sees in her daily life.

A strength and limitation of this work is that we used existing warning labels. While this allowed us to examine genuine awareness, we were constricted to use labels currently appearing on cigarette packages in the countries where we were doing data collection. As a result, we tested a heterogeneous group of warning labels. In future and experimental studies, it would be valuable to control features and assess the children’s awareness and understanding associated with specific label features such as text type, image graphicness/gruesomeness, and message abstraction. Such research, albeit artificial, would offer information on effectiveness of different types of warning labels.

From this and other research, it is known that for health messages to be effective (influence attitudes and behaviors), message receivers must first be exposed, attend to, and elaborate on the presented information (Petty and Priester 1994). Message receivers must understand the communication before they can accept and act on the messages (Wilson 2007; Petty and Cacioppo 1986). For the youngest audience groups, there is a preference for simple graphics and text; visual complexity, advanced language, and complex analogies will impede recall and comprehension (Peracchio and Luna 1998; Watt and Welch 1984). In some cases, children will even seek to avoid the messages if they are too difficult or frightening to process (Witte and Allen 2000; Wartella and Ettema 1974). Additionally, this study used the less gruesome labels, so we were unable to examine if more frightening messages lead to more or less awareness.

Young children, given their developing abilities around information processing, can be a difficult group to reach with health messages (Wilson 2007). As noted earlier, it is critical that health communication directed towards young children be simple, explicit, and avoid abstractions (Peracchio and Luna 1998). According to Article 11 of the WHO’s FCTC, health warning labels are trying to communicate the health risks associated with smoking to a range of subgroups. A challenge for developers is to create, in a single message, information that is valuable to diverse audiences, from the young non-smoker, to the occasional smoker, to the regular smoker looking towards cessation (Flynn et al. 2007; Strahan et al. 2002). In this study we observed the greatest understanding where the labels featured overt, direct consequences of smoking (Brazil and Pakistan). In India, where a scorpion is featured on the warning label, under 2 % of the children showed solid understanding of the warning label message. Text in contrast may offer a clear message (i.e., “Smoking Kills”); however, such messages require a child to be literate.

In most countries, cigarette packages now feature health warning labels, but these vary in the text and graphic messages used. Labels can be a direct and prominent way to communicate to different populations (Hammond 2011), but this study suggests warning labels are reaching a very small percentage of young children. Even among those who had seen the labels, most had either no or weak understanding of what these labels were trying to convey. Without awareness or understanding, it is unlikely that cigarette health-warning labels will have an impact on this (or any) age group. As youth are among the subpopulations specified by the WHO’s FCTC, it is important that children and adolescents are better reached with these health warnings and messages (WHO 2012). Naturalistic studies such as this one, and experimental studies examining specific features of warning labels, must continue in order to develop effective messages that reach and inform different audiences about the health risks associated with tobacco use.

## References

[CR1] Borland R, Yong HH, Wilson N, Font GT, Hammond D, Cumming KM, Hosking W, McNeill A (2009). How reactions to cigarette packet health warnings influence quitting: findings from the ITC Four-Country survey. Addiction.

[CR2] Borzekowski DLG, Cohen JE (2013). International reach of tobacco marketing among young children. Pediatrics.

[CR3] Borzekowski DLG, Macha J (2010). The role of *Kilimani Sesame* in the healthy development of Tanzanian preschool children. J Appl Dev Psychol.

[CR4] Borzekowski D, Clearfield E, Rimal R, Gielen AC (2013) Young children’s perceptions of fire-safety messages: Do framing and parental mediation matter? J Burn Care Res. doi.10.1097/BCR.0b013e31829afe6c 10.1097/BCR.0b013e31829afe6c 23877137

[CR5] Breslau N, Pereson EL (1996). Smoking cessation in young adults: age at initiation of cigarette smoking and other suspected influences. AJPH.

[CR6] DiFranza JR, Wellman RJ, Sargent JD, Weitzman M, Hipple BJ (2006). Tobacco promotion and the initiation of tobacco use: assessing the evidence for causality. Pediatrics.

[CR7] Emria S, Bagcib T, Karakocaa Y, Barisc E (1998). Recognition of cigarette brand names and logos by primary schoolchildren in Ankara, Turkey. Tob Control.

[CR8] Flynn BS, Worden JK, Bunn JY, Dorwaldt AL, Connolly SW, Ashikaga T (2007). Youth audience segmentation strategies for smoking-prevention mass media campaigns based on message appeal. Health Educ Behav.

[CR9] Fong GT (2007) The International Tobacco Control Policy Evaluation Project: evaluating global tobacco policies of the Framework Convention on Tobacco Control. In: Proc. of 8th Asia Pacific Conference on Tobacco or Health (APACT) Taipei, Taiwan, October 2007

[CR10] Freeman D, Brucks M, Wallendorf M (2005). Young children’s understandings of cigarette smoking. Addiction.

[CR11] Gilpin EA, Pierce JP (1997). Trends in adolescent smoking initiation in the United States: Is tobacco marketing an influence?. Tob Control.

[CR12] Gilpin EA, Lee L, Pierce JP (2004). Does adolescent perception of difficulty in getting cigarettes deter experimentation?. Prev Med.

[CR13] Gilpin EA, White MM, Messer K, Pierce JP (2007). Receptivity to tobacco advertising and promotions among young adolescents as a predictor of established smoking in young adulthood. AJPH.

[CR14] Global Youth Tobacco Survey Collaborative Group (2002). Tobacco use among youth: a cross country comparison. Tob Control.

[CR15] Goodall C, Appiah O (2008). Adolescents’ perceptions of Canadian cigarette package warning labels: investigating the effects of the message framing. Health Commun.

[CR16] Hammond D (2011). Health warning messages on tobacco products: a review. Tob Control.

[CR17] Hammond D (No Date) Health warning images: tobacco labeling regulations. http://www.tobaccolabels.ca/healthwarningimages. Accessed 2 August 2013

[CR18] Jha P, Ranson K, Nguyen SN, Yach D (2002). Estimates of global and regional smoking prevalence by age and sex. AJPH.

[CR19] Kessels LTE, Ruiter RAC (2012). Eye movement responses to health messages on cigarette packages. BMC Public Health.

[CR20] Koh HK, Alpert HR, Judge CM, Caughey RW, Elqura LJ, Connolly G, Warren CW (2011). Understanding worldwide youth attitudes toward smoke-free policies: an analysis of the Global Youth Tobacco Survey. Tob Control.

[CR21] Leonardi-Bee J, Jere ML, Britton J (2011). Exposure to parental and sibling smoking and the risk of smoking uptake in childhood and adolescence: a systematic review and meta-analysis. Thorax.

[CR22] Partos TR, Borland R, Yong HH, Thrasher J, Hammond D (2013). Cigarette packet warning labels can prevent relapse: findings from the International Tobacco Control 4-Country policy evaluation cohort. Tob Control.

[CR23] Peracchio LA, Luna D (1998). The development of an advertising campaign to discourage smoking initiation among children and youth. J Adv.

[CR24] Petty RE, Cacioppo JT (1986). Communication and persuasion: central and peripheral routes to attitude change.

[CR25] Petty RE, Priester JR, Bryant J, Zillmann D (1994). Mass media attitude change: implications of the Elaboration Likelihood model of persuasion. Media effects.

[CR26] Selin H (2009). Tobacco packaging and labeling: technical guide.

[CR27] Shafey O, Eriksen M, Ross H, Mackay J (2009). The tobacco atlas.

[CR28] Shanahan P, Elliott D (2009). Evaluation of the effectiveness of the graphic health warnings on tobacco product packaging 2008.

[CR29] Smith KH, Stutts MA (2003). Effects of short-term cosmetic versus long-term health fear appeals in anti-smoking advertisements on the smoking behavior of adolescents. J Consum Behav.

[CR30] StataCorp (2009). Stata statistical software: release 11.

[CR31] Strahan EJ, White K, Fong GT, Fabrigar LR, Zanna MP, Cameron R (2002). Enhancing the effectiveness of tobacco package warning labels: a social psychological perspective. Tob Control.

[CR32] Thrasher JF, Villalobos V, Szklo A, Fong GT, Perez C, Sebrie E, Sansone N, Figueiredo V, Boado M, Arillo-Santillan E, Bianco E (2010). Assessing the impact of cigarette package health warning labels: a cross-country comparison in Brazil, Uruguay and Mexico. Salud Publica Mex.

[CR33] Wartella E, Ettema JA (1974). A cognitive developmental study of children’s attention to television commercials. Comm Res.

[CR34] Watt JH, Welch AJ, Bryant J, Anderson DR (1984). Effects of static and dynamic complexity on children’s attention and recall of televised instruction. Children’s understanding of television.

[CR35] Wilson BJ (2007). Designing media messages about health and nutrition: What strategies are most effective?. J Nutr Educ Behavior.

[CR36] Witte K, Allen M (2000). A meta-analysis of fear appeals: implications for effective public health campaigns. Health Educ Behav.

[CR37] World Health Organization (WHO) (2008) Guidelines for implementation of Article 11 of the WHO Framework Convention on Tobacco Control (packaging and labeling of tobacco products). http://www.who.int/fctc/protocol/guidelines/adopted/article_11/en/index.html. Accessed 21 February 2013

[CR38] World Health Organization (WHO) (2012) World Health Organization Country Statistics. www.who.int/countries. Accessed 31 October 2012

